# Modeling natural coinfection in a bat reservoir shows modulation of Marburg virus shedding and spillover potential

**DOI:** 10.1371/journal.ppat.1012901

**Published:** 2025-03-17

**Authors:** Amy J. Schuh, Brian R. Amman, Jonathan C. Guito, James C. Graziano, Tara K. Sealy, Jonathan S. Towner

**Affiliations:** 1 Viral Special Pathogens Branch, Division of High-Consequence Pathogens and Pathology, National Center for Emerging and Zoonotic Infectious Diseases, United States Centers for Disease Control and Prevention, Atlanta, Georgia, United States of America; 2 United States Public Health Service Commissioned Corps, Rockville, Maryland, United States of America; The Ohio State University, UNITED STATES OF AMERICA

## Abstract

The Egyptian rousette bat (ERB) is a natural reservoir for Marburg virus (MARV; family *Filoviridae*), a putative reservoir for Sosuga virus (SOSV; family *Paramyxoviridae*), and a vertebrate reservoir for Kasokero virus (KASV; family *Orthonairoviridae*); however, the effect of naturally occurring coinfection by those viruses on MARV shedding and spillover potential is unknown. To answer this question, we experimentally infected one cohort of captive-bred ERBs with SOSV+MARV (n=12 bats) or MARV only (n=12 bats) and a second cohort with KASV+MARV (n=12 bats) or MARV only (n=12 bats), and then collected blood, oral swab, and rectal swab specimens throughout the course of infection to monitor viral shedding. Compared to the MARV-monoinfected bat group, the SOSV+MARV-coinfected bat group exhibited a significantly shortened duration of MARV oral shedding and a significantly decreased anti-MARV IgG response, which may increase the capacity for MARV reinfection. In contrast, relative to the MARV-monoinfected bat group, the KASV+MARV-coinfected bat group exhibited significantly increased peak magnitudes and durations of MARV viremia and oral shedding, as well as a significantly increased anti-MARV IgG response. Correspondingly, cumulative MARV shedding loads, a measure of infectiousness, were significantly higher in the KASV+MARV-coinfected bat group than the MARV-monoinfected bat group. Four of the KASV+MARV-coinfected bats were classified as MARV supershedders, together accounting for 72.5% of the KASV-MARV experimental cohort’s total shedding. Our results demonstrate that SOSV+MARV and KASV+MARV coinfection of ERBs differentially modulates MARV shedding and anti-MARV IgG responses, thereby implicating MARV coinfection as playing a critical role in bat-to-bat MARV transmission dynamics and spillover potential.

## Author summary

Zoonotic pathogen spillover from wildlife reservoirs to humans has initiated numerous epidemics throughout history, resulting in significant mortality and devastating economic consequences. However, there is a lack of knowledge on the impact that naturally occurring ecological stressors of wildlife, such as coinfection, have on pathogen shedding and zoonotic spillover potential. Addressing this knowledge gap is crucial for developing effective strategies to prevent or mitigate the effect of zoonotic pathogen spillover from wildlife and future epidemics. The Egyptian rousette bat (ERB) is a natural reservoir for Marburg virus (MARV), the putative reservoir for Sosuga virus (SOSV), and a vertebrate reservoir for Kasokero virus (KASV). Here, we reconstituted naturally occurring coinfection by experimentally infecting groups of 12 ERBs with SOSV+MARV, KASV+MARV, or MARV only. We found that SOSV+MARV coinfection decreased the duration of MARV oral shedding, while KASV+MARV coinfection increased the duration and intensity of MARV viremia and oral shedding. Additionally, we showed that cumulative MARV shedding loads, a measure of infectiousness, were higher in KASV+MARV-coinfected bats than MARV-monoinfected bats and four of the KASV+MARV-coinfected bats were identified as MARV supershedders. Our findings demonstrate that, depending on the specific coinfecting virus, MARV shedding is differentially modulated, thereby affecting spillover potential.

## Introduction

Zoonotic pathogen spillover from wildlife to humans has initiated numerous epidemics throughout history, including human immunodeficiency virus (HIV) [1], Ebola virus [2], and severe acute respiratory syndrome coronavirus 1 (SARS-CoV-1) [3]. These epidemics have resulted in significant mortality (e.g., an estimated 42.3 million people have died of HIV since the beginning of the epidemic [4]), excess deaths from other causes (e.g., an estimated 10,600 excess lives were lost to HIV, tuberculosis, and malaria due to a reduction of other healthcare services in Guinea, Liberia, and Sierra Leone in 2015 during the 2014─2016 Ebola outbreak [5]), and devastating economic losses (e.g., global economic loss from SARS-CoV-1 was an estimated $40 billion US dollars in 2003) [6], underscoring the need to identify ecological factors that increase the risk of zoonotic spillover. Identification of factors that increase the risk of pathogen transmission from wildlife to humans will help inform evidence-based mitigation strategies to prevent zoonotic pathogen spillover and future epidemics [7].

The probability of zoonotic pathogen spillover is determined by several interacting factors [8] including the dynamics of infection in reservoir hosts [9], microbiological determinants of pathogen survival outside of reservoir hosts [10], epidemiological and behavioral determinants of exposure [11], and factors that influence human susceptibility to infection [12]. The former of which is influenced by the density of reservoir hosts, as well as the prevalence and intensity of infection. Factors such as reservoir hosts’ nutritional status and the presence of more than one infectious agent can modulate the prevalence and intensity of infection. Previous studies, primarily focusing on humans, have demonstrated that the interactions of coinfecting microorganisms within the host can result in synergistic, antagonistic, or neutral outcomes [13]. Synergistic outcomes of coinfection have been shown to increase host shedding (e.g., herpes simplex virus type 2 genital shedding was 3.7 times greater in HIV seropositive women than HIV seronegative women [14]), morbidity (e.g., mean time from hepatitis C virus (HCV) infection to cirrhosis was 16.3 years faster in HIV positive patients than HIV negative patients [15]), and mortality (e.g., HIV positive patients who had progressed to acquired immunodeficiency syndrome (AIDS) and were coinfected with hepatitis B virus (HBV) or HCV had a 51.7% reduction in survival time compared to AIDS patients that were not coinfected with HBV or HCV [16]). While antagonistic outcomes have been shown to protect against infection (e.g., human rhinovirus infection reduced the likelihood of influenza A virus (IAV) infection by 86% [17], decrease morbidity (e.g., individuals coinfected with *Giardia lamblia* parasites and rotavirus had a 27.3% reduction in the severity of diarrheal episodes compared to individuals infected with rotavirus alone [18]), and decrease mortality (e.g., individuals coinfected with HIV and human pegivirus exhibited slower progression to AIDS and a reduced mortality rate compared to those infected with HIV only [19]). Although much research has been devoted to understanding the outcomes of coinfection in humans and numerous studies have reported that wildlife are often coinfected with microorganisms that are likely pathogenic to humans [20–22], there is a lack of information on the impact that coinfection of wildlife reservoir hosts has on pathogen shedding dynamics, infectiousness, and zoonotic spillover potential.

Egyptian rousette bats (ERBs; *Rousettus aegyptiacus*) are distributed throughout sub-Saharan Africa and often found inhabiting subterranean environments with populations exceeding 100,000 individuals [23]. ERBs are natural reservoirs (species in which a pathogen is permanently maintained) [24] for highly-pathogenic Marburg virus (family *Filoviridae*, genus *Orthomarburgvirus*; cousin to Ebola virus) [25–28], putative natural reservoirs (species in which a pathogen is thought to be maintained; scientific evidence is not definitive but strongly suggestive) [24] for Sosuga virus (family *Paromyxoviridae*, genus *Pararubalavirus*; cousin to Nipah virus) [29–31], and natural vertebrate reservoirs (vertebrate species in which a vector-borne pathogen is permanently maintained and/or amplified) [32] for Kasokero virus (family *Orthonairoviridae*, genus *Orthonairovirus*; cousin to Crimean-Congo hemorrhagic fever virus) [33–35]. MARV causes outbreaks of hemorrhagic disease in sub-Saharan Africa that are characterized by human-to-human transmission and high case fatality ratios (mean: 80.1%) [36]. A longitudinal investigation of orthomargburgvirus infection in ERBs inhabiting Python Cave and Kitaka Mine, Uganda following a series of MARV spillover events linked to these locations [37,38] revealed a seasonal, age-associated pattern of virus infection [27]. Maternal orthomarburgvirus antibodies likely protect ERB pups from infection (0.0% PCR prevalence) until they become independent juveniles at ~3-months of age (2.7% PCR prevalence) [27]. The prevalence of active orthomarburgvirus infection peaks in 6-month-old ERB juveniles (12.4% PCR prevalence), coincidental with the timing of the biannual birthing seasons, and then declines to a consistent, year-round prevalence by the time the juveniles become adults at 7─8 months of age (2.4% PCR prevalence) [27]. Subsequent experimental studies using MARV-experimentally inoculated, captive ERBs revealed that the virus is primarily shed in oral secretions but can also be detected in blood, rectal swabs, and urine [28,39–41], and an experimental transmission study demonstrated horizontal transmission of MARV from infected to naïve contact ERBs [41]. SOSV was first described after the virus was isolated from a person that became severely ill after being in direct and indirect contact with bats in South Sudan and Uganda [29]. The virus was subsequently detected in multiple tissues, rectal swabs, and oral swabs from ERBs captured in Uganda [30] and Sierra Leone [42] but infectious virus has yet to be isolated from wild-caught ERBs. Experimental infection of captive-bred ERBs with SOSV revealed that infectious virus is primarily shed in the feces but can also be detected in blood, oral swabs, and urine [31]). KASV was discovered in 1977 after 3 scientists involved in the isolation and characterization of KASV from ERB serum samples contracted the virus (and 1 taxi driver) and presented with clinical manifestations ranging in severity from a mild febrile illness to a long-lasting systemic disease [33]. Infectious KASV was later isolated from engorged and unengorged *Ornithodoros* (*Reticulinasus*) *faini* ticks collected from rock crevices of ERB roosts in Lanner Gorge Cave, South Africa and Python Cave, Uganda [34]. Experimental infection of captive-bred ERBs with KASV revealed that the virus is primarily detected in the blood but can also be shed in oral swabs, rectal swabs, and urine [35,43]. Although longitudinal investigations of SOSV and KASV infection in natural ERB colonies are lacking, we expect, like MARV, the prevalence of active SOSV and KASV infection peaks in 6-month-old juveniles, leading to a spike in individuals coinfected with MARV, SOSV, and/or KASV.

In this study, we assessed the impact of virus coinfection on MARV shedding, and reinfection and spillover potential by comparing high-resolution viremia, oral shedding, fecal shedding, and anti-MARV IgG antibody response data from captive-bred ERBs coinfected with SOSV+MARV or KASV+MARV to ERBs infected with MARV only. We demonstrated that coinfection differentially modulates MARV shedding, MARV bat-to-bat transmission dynamics, and zoonotic spillover potential by showing that SOSV+MARV coinfection of ERBs modestly inhibits MARV shedding and decreases anti-MARV IgG responses, while KASV+MARV coinfection of ERBs significantly increases MARV shedding and infectiousness, as well as anti-MARV IgG antibody responses.

## Results

### General notes

**Fig 1:**
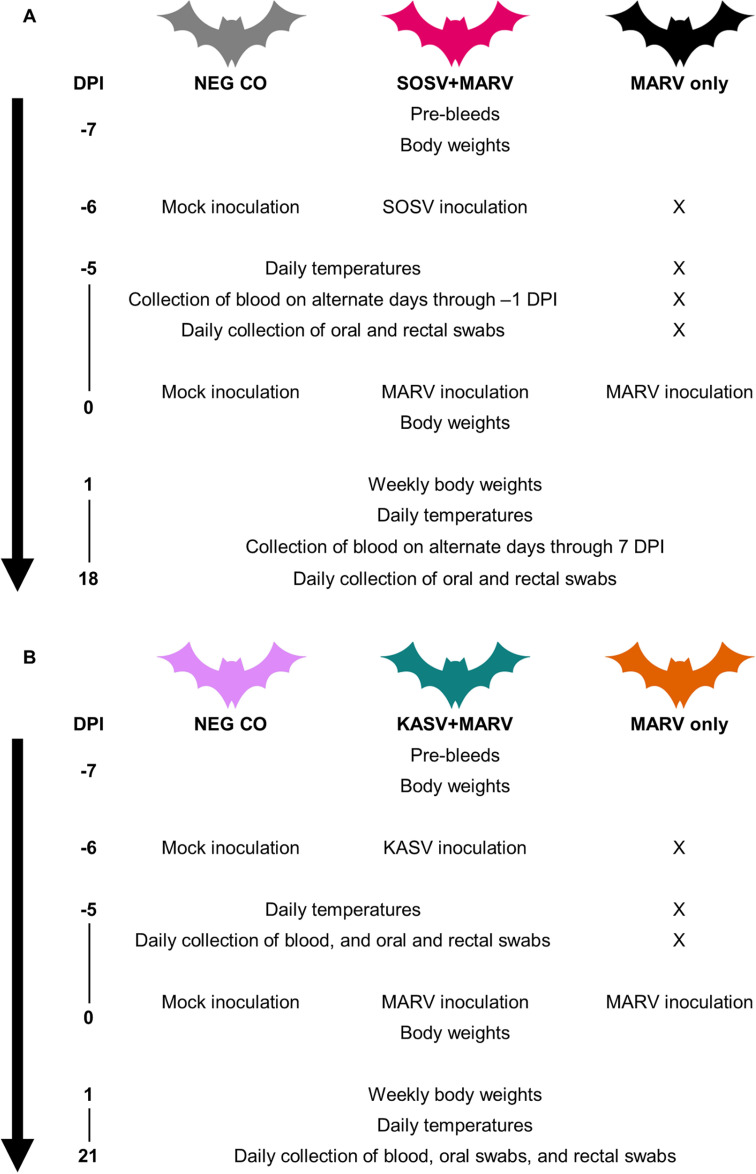
Experimental design. (A) SOSV-MARV experimental cohort and (B) KASV-MARV experimental cohort. DPI: Days post infection (relative to MARV inoculation). X: Procedure not performed.

At -6 days post MARV inoculation, 2 negative control (NEG CO) bats and 12 SOSV+MARV bats in the SOSV-MARV experimental cohort were inoculated with sterile media and SOSV, respectively (Fig 1A). Six days later at 0 days post MARV infection, the NEG CO bats were inoculated with sterile media, while the SOSV+MARV bats and 12 MARV only bats were inoculated with MARV. Similarly, at -6 days post MARV infection, 2 NEG CO bats and 12 KASV+MARV bats in the KASV-MARV experimental cohort were inoculated with sterile media and KASV, respectively (Fig 1B). Six days later at 0 days post MARV infection, the NEG CO bats were inoculated with sterile media, while the KASV+MARV bats and 12 MARV only bats were inoculated with MARV. For bats in the SOSV-MARV experimental cohort, blood was collected every other day through 7 days post MARV inoculation, and oral swabs and rectal swabs were collected daily through 18 days post MARV inoculation. For bats in the SOSV-MARV experimental cohort, blood, oral swabs, and rectal swabs were collected daily through 21 days post MARV inoculation.

Throughout the study, none of the bats in the SOSV-MARV or KASV-MARV experimental cohorts exhibited overt signs of clinical illness, and weights (S1A and S1B Fig) and temperatures (S1C and S1D Fig) were consistent with what has previously been observed for ERBs experimentally monoinfected with SOSV [31], MARV [28,39–41], and KASV [35,43]. It should be noted that single daily temperature measurement may not capture short-lived or intermittent post-inoculation febrile responses. Viral loads in blood, oral, and rectal specimens collected from the bats were measured by RT-qPCR and are reported hereafter as log_10_ 50% tissue culture infectious dose equivalents ml^-1^ (log_10_TCID_50_eq ml^-1^). All specimens collected from NEG CO bats in the SOSV-MARV and KASV-MARV experimental cohorts tested uniformly negative for MARV, SOSV, and KASV RNA. Likewise, all specimens collected from SOSV- MARV-, and/or KASV-inoculated bats tested uniformly negative for viruses other than the inoculum(s), indicating the absence of virus cross-contamination between bat groups.

### SOSV+MARV coinfection modestly inhibits MARV shedding

**Fig 2:**
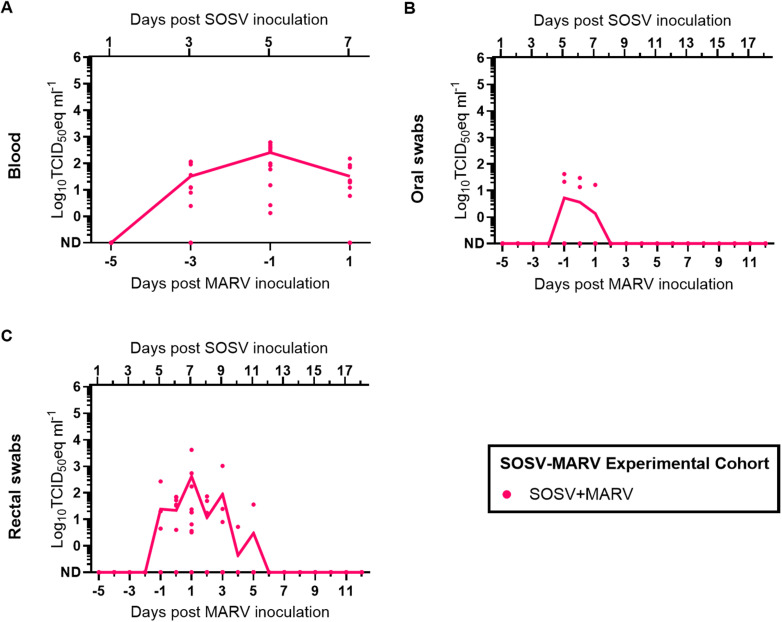
SOSV loads in specimens obtained from bats in the SOSV-MARV experimental cohort. RT-qPCR-derived log_10_TCID_50_eq ml^-1^ SOSV loads in **(A)** blood, **(B)** oral swabs, and **(C)** rectal swabs. Days post MARV infection is shown for reference only. Symbols in **A**-**C** represent individual bats, and solid lines represent arithmetic mean viral loads. ND: Not detected.

**Fig 3:**
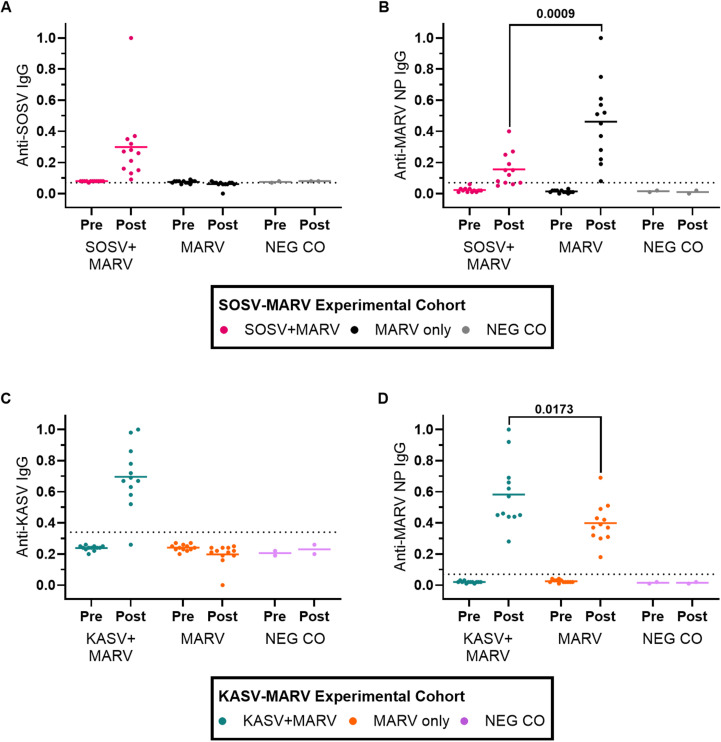
Pre- and post-study virus-specific IgG responses for bats in the SOSV-MARV and KASV-MARV experimental cohorts. (A) Anti-SOSV IgG responses for bats in the SOSV-MARV experimental cohort, **(B)** anti-MARV nucleoprotein (NP) IgG responses for bats in the SOSV-MARV experimental cohort, **(C)** anti-KASV IgG responses for bats in the KASV-MARV experimental cohort, and **(D)** anti-MARV NP IgG responses for bats in the KASV-MARV experimental cohort. Post-study whole blood specimens were collected on the same day post MARV inoculation for all bats in the SOSV-MARV experimental cohort (day 18) and for all bats in the KASV-MARV experimental cohort (day 21). Symbols in **A**-**D** represent individual bats, solid lines represent arithmetic means, and dotted horizontal lines represent the cut-off values of the serological assays. Post-study whole blood specimens were collected on different days post SOSV infection for bats in **A** and different days post KASV infection for bats in **C**, and are therefore not comparable. The Shapiro-Wilks test determined that post-study anti-MARV NP IgG response datasets for coinfected and monoinfected bats in **B** and **D** are normally-distributed. Therefore, unpaired t-tests were used to determine if mean anti-MARV NP IgG significantly differed (P<0.05) between coinfected bats (n=12) and monoinfected bats (n=12). P values are located above each comparison and statistically significant values are bolded.

Evidence of SOSV infection was detected in all SOSV+MARV bats in at least 1 blood specimen (12/12 bats; Fig 2A), oral swab (3/12 bats; Fig 2B), or rectal swab (10/12 bats; Fig 2C) collected during the study. At the end of the study (18 days post MARV inoculation; 24 days post SOSV inoculation), 11/12 of the SOSV+MARV bats had seroconverted to SOSV (Fig 3A).

**Fig 4:**
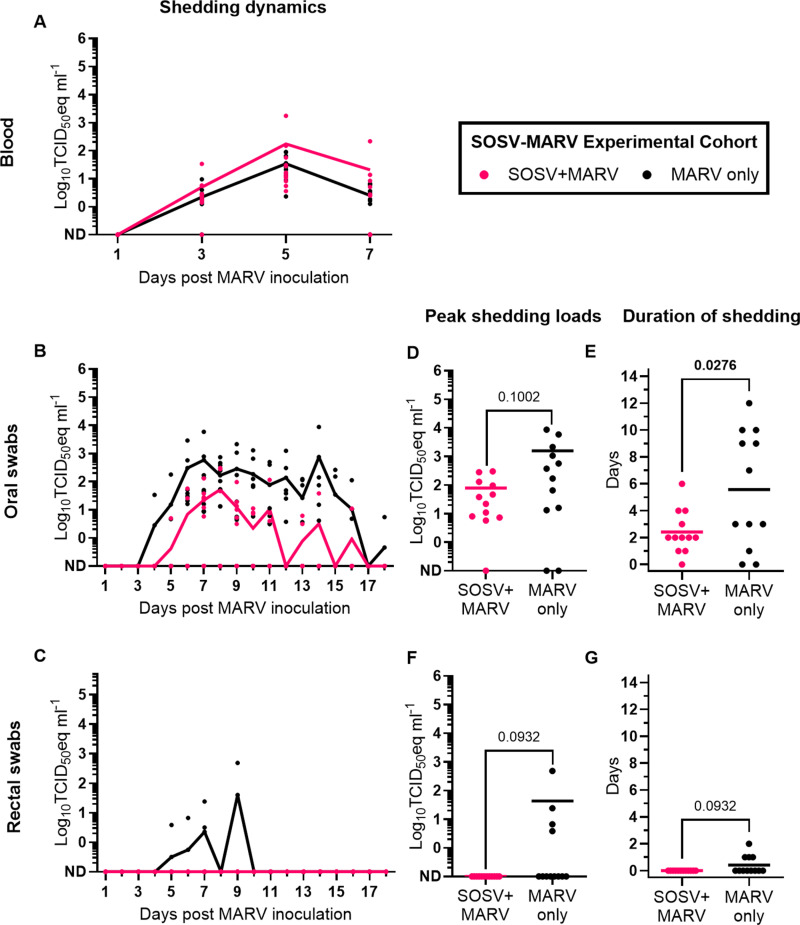
MARV shedding dynamics in bats from the SOSV-MARV experimental cohort. RT-qPCR-derived log_10_TCID_50_eq ml^-1^ MARV loads in **(A)** blood, **(B)** oral swabs, and **(C)** rectal swabs. Distribution of **(D)** peak oral MARV shedding loads, **(E)** the duration of oral MARV shedding, **(F)** peak MARV rectal shedding loads, and **(G)** the duration of rectal MARV shedding. Symbols in **A-G** represent individual bats, and solid lines represent arithmetic means. The Shapiro-Wilks test determined that the datasets represented in **D**, **F**, and **G** do not follow a normal or lognormal distribution, and the dataset represented in **E** is normally distributed. Therefore, Mann-Whiney U tests were used to determine if peak shedding loads (**D** and **F**) and the duration of shedding **(G)** significantly differed (P<0.05) between coinfected bats (n=12) and monoinfected bats (n=12), while an unpaired t-test was used to determine if the duration of shedding significantly differed between coinfected and monoinfected bats. P values are located above each comparison and statistically significant values are bolded. ND: Not detected.

MARV RNA was detected in all SOSV+MARV bats and MARV only bats in at least 1 blood specimen (12/12 SOSV+MARV bats and 12/12 MARV only bats; Fig 4A), oral swab (11/12 SOSV+MARV bats and 10/12 MARV only bats; Fig 4B), or rectal swab (0/12 SOSV+MARV bats and 4/12 MARV only bats; Fig 4C) collected during the study. Although peak oral shedding loads did not significantly differ between SOSV+MARV bats and MARV only bats (Fig 4D), the duration of MARV oral shedding was 3.2 days shorter (unpaired-t=2.359, df=22, P=0.0276; Fig 4E) in SOSV+MARV bats (mean=2.4 days, SD=1.6) compared to MARV only bats (mean=5.6 days, SD=4.4). Neither peak MARV fecal shedding loads (Fig 4F) nor the duration of MARV fecal shedding (Fig 4G) significantly differed between SOSV+MARV bats and MARV only bats. At the end of the study (18 days post MARV inoculation), 10/12 of the SOSV+MARV bats and 12/12 of the MARV only bats had seroconverted to MARV (Fig 3B). Post-study anti-MARV nucleoprotein (NP) IgG responses were significantly lower (unpaired t=3.817, df=22, P=0.0009) in MARV+SOSV bats (mean adjusted sum OD=0.16, SD=0.11) than MARV only bats (mean adjusted sum OD=0.46, SD=0.26).

### KASV+MARV coinfection significantly potentiates MARV shedding

**Fig 5:**
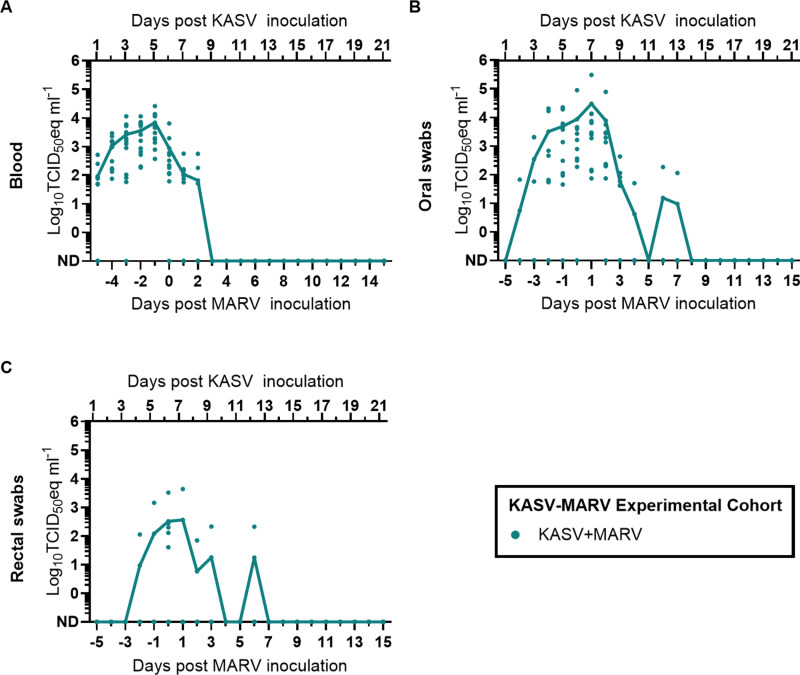
KASV loads in specimens obtained from bats in the KASV-MARV experimental cohort. RT-qPCR-derived log_10_TCID_50_eq ml^-1^ SOSV loads in **(A)** blood, **(B)** oral swabs, and **(C)** rectal swabs. Days post MARV infection is shown for reference only. Symbols in **A-C** represent individual bats, and solid lines represent arithmetic mean viral loads. ND: Not detected.

Evidence of KASV infection was detected in all KASV+MARV bats in at least 1 blood specimen (12/12 bats; Fig 5A), oral swab (12/12 bats; Fig 5B), or rectal swab (7/12 bats; Fig 5C) collected during the study. At the end of the study (21 days post MARV inoculation; 27 days post KASV inoculation), 11/12 of the KASV+MARV bats had seroconverted to KASV (Fig 3C).

**Fig 6:**
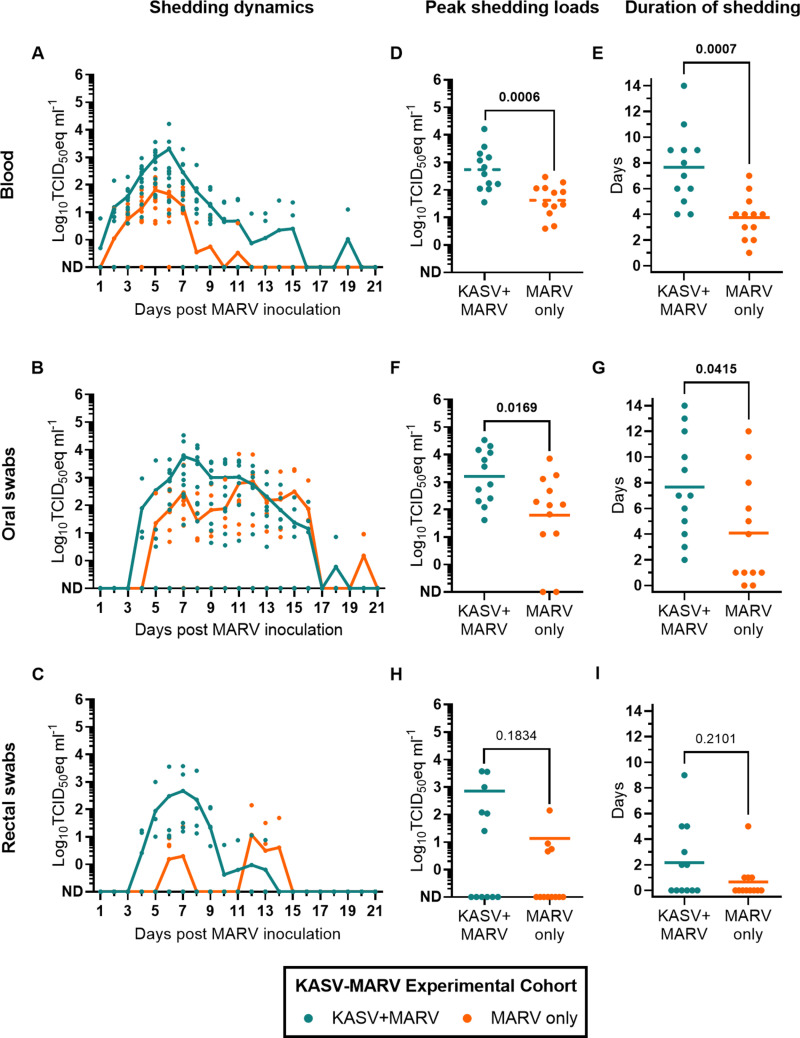
MARV shedding dynamics in bats from the KASV-MARV experimental cohort. RT-qPCR-derived log_10_TCID_50_eq ml^-1^ MARV loads in **(A)** blood, **(B)** oral swabs, and **(C)** rectal swabs. Distribution of **(D)** peak MARV viremias, **(E)** the duration of MARV viremia, **(F)** peak oral MARV shedding loads, **(G)** the duration of oral MARV shedding, **(H)** peak MARV rectal shedding loads, and **(I)** the duration of rectal MARV shedding. Symbols in **A-I** represent individual bats, solid lines represent arithmetic means, and dashed lines in **D** represent geometric means. The Shapiro-Wilks test determined that the dataset represented in **D** is lognormally distributed, the dataset represented in **G** is normally-distributed, and the datasets represented in **E**, **F**, **H**, and **I** do not follow a normal or lognormal distribution. Therefore, to determine if peak shedding and the duration of shedding significantly differed (P<0.05) between coinfected (n=12) and monoinfected (n=12) bats, an unpaired t-test was applied to the log-transformed dataset represented in **D**, an unpaired t-test was applied to the dataset represented in **G**, and Mann-Whiney U tests were applied to the datasets represented in **E**, **F**, **H**, and **I.** P values are located above each comparison and statistically significant values are bolded. ND: Not detected.

MARV RNA was detected in all KASV+MARV bats in at least 1 blood specimen (12/12 bats; Fig 6A), oral swab (12/12 bats; Fig 6B), or rectal swab (6/12 bats; Fig 6C) collected during the study. Peak MARV viremia loads were 0.6 log_10_TCID_50_eq ml^-1^ higher (t=3.970, df=22, P=0.0006; Fig 6D) in KASV+MARV bats than MARV only bats, and the duration of MARV viremia was 3.9 days longer (t=3.963, df=22, P=0.0007) in KASV+MARV bats (mean=7.7, SD=3.0) than MARV only bats (mean=3.8, SD=1.7; Fig 6E). Peak MARV oral shedding loads were 0.9 log_10_TCID_50_eq ml^-1^ higher (Mann-Whitney U=31, P=0.0169; Fig 6F) in KASV+MARV bats (mean=3.9 log_10_TCID_50_eq ml^-1^, SD=4.0) compared to MARV only bats (mean=3.0 log_10_TCID_50_eq ml^-1^, SD=3.3) and the duration of MARV oral shedding was 3.6 days longer (t=2.165, df=22, P=0.0415; Fig 6G) in KASV+MARV bats (mean=7.7 days, SD=4.0) compared to MARV only bats (mean=4.1 days, SD=4.1). Neither peak MARV fecal shedding loads (Fig 6H) nor the duration of MARV fecal shedding (Fig 6I) significantly differed between KASV+MARV and MARV only bats. At the end of the study (21 days post MARV inoculation), all of the KASV+MARV bats and MARV only bats had seroconverted to MARV (Fig 3B). Post-study anti-MARV NP IgG responses were significantly higher (unpaired t=2.573, df=22, P=0.0173) in MARV+KASV bats (mean adjusted sum OD=0.58, SD=0.21) than MARV only bats (mean adjusted sum OD=0.40, SD=0.13).

### Coinfection modulates shedding and therefore infectiousness

**Fig 7:**
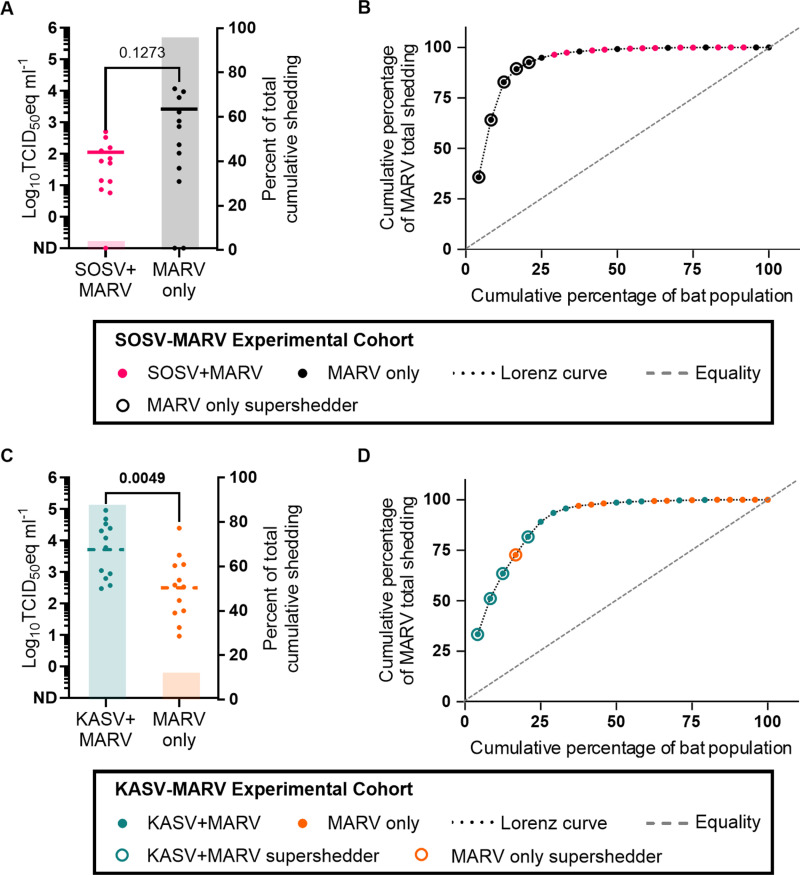
Cumulative MARV shedding in bats from the SOSV-MARV and KASV-MARV experimental cohort. (A) RT-qPCR-derived log_10_TCID_50_eq ml^-1^ MARV cumulative shedding loads (sum of viral loads detected in oral swabs and rectal swabs from 0-18 days post MARV inoculation) for bats from the SOSV-MARV experimental cohort are denoted by symbols (solid lines represent arithmetic means) and represented on the left-y axis, and the percent of total cumulative shedding according to group are denoted by bars and represented on the right-y axis. (B) Lorenz curve of cumulative percentage of the SOSV-MARV experimental cohort bat population versus cumulative percentage of MARV shedding ranked in descending order (e.g., first symbol on the curve represents the bat which had the highest cumulative percentage of MARV shedding). (C) RT-qPCR-derived log_10_TCID_50_eq ml^-1^ MARV cumulative shedding loads (sum of viral loads detected in blood, oral swabs, and rectal swabs from 0-21 days post MARV inoculation) for bats from the KASV-MARV experimental cohort are denoted by symbols (dotted lines represent geometric means) and represented on the left-y axis, and the percent of total cumulative shedding according to group are represented by bars and shown on the right-y axis. (D) Lorenz curve of cumulative percentage of the KASV-MARV experimental cohort bat population versus cumulative percentage of MARV shedding ranked in descending order. The Shapiro-Wilks test determined that the cumulative shedding load dataset represented in **A** does not follow a normal or lognormal distribution and the cumulative shedding load dataset in **C** is lognormally-distributed. Therefore, to determine if cumulative shedding loads significantly differed (P<0.05) between coinfected (n=12) and monoinfected (n=12) bats, a Mann-Whiney U test was applied to the dataset represented in **A** and an unpaired t-test was applied to the log-transformed dataset represented in **C**. P values are located above each comparison and statistically significant values are bolded. ND: Not detected.

Cumulative viral shedding is a proxy for infectiousness [44] and was assessed to determine the impact of viral coinfection on MARV infectiousness (bat-to-bat and bat-to-human transmission potential) by summing viral RNA loads detected in blood (KASV-MARV experimental cohort only), oral swabs, and rectal swabs throughout the study. Although cumulative MARV shedding loads were lower in SOSV+MARV bats (mean=2.0 log_10_TCID_50_eq ml^-1^, SD=2.2; contributed 4.1% of the SOSV-MARV experimental cohort’s total cumulative MARV shedding) than MARV only bats (mean=3.4 log_10_TCID_50_eq ml^-1^, SD=3.6; contributed 95.9% of the SOSV-MARV experimental cohort’s total cumulative MARV shedding), the difference was not statistically significant (Mann-Whitney U=45, P=0.1273; Fig 7A). Nevertheless, the Lorenz curve of cumulative percentage of the SOSV-MARV experimental cohort’s bat population versus cumulative percentage of MARV shedding shows that a minority of the experimental cohort population contributed to a disproportionately large percentage of MARV shedding (Fig 7B). Of the 24 bats in the SOSV-MARV experimental cohort, 5 of the MARV only bats were classified as supershedders as they each shed levels greater than the 80^th^ percentile and together accounted for 92.6% of the experimental cohort’s cumulative MARV shedding.

For the KASV-MARV experimental cohort, cumulative MARV shedding loads were 1.2 log_10_TCID_50_eq ml^-1^ higher (unpaired t=3.126, df=22, P=0.0049; Fig 7C) in KASV+MARV bats (geometric mean=3.7, geometric SD=0.9) than MARV only bats (geometric mean=2.5, geometric SD=1.0), with the KASV+MARV bats contributing to 87.8% of the experimental cohort’s cumulative MARV shedding load. Correspondingly, the Lorenz curve shows that a minority of the KASV-MARV experimental cohort contributed to a disproportionately large percentage of MARV shedding (Fig 7D). Of the 24 bats in the KASV-MARV experimental cohort, 4 of the KASV+MARV bats were classified as MARV supershedders, while only 1 of the MARV only bats was classified as a MARV supershedder. The 4 KASV+MARV supershedder bats accounted for 72.5% of the KASV-SOSV experimental cohort’s cumulative MARV shedding.

## Discussion

This study demonstrates that SOSV+MARV and KASV+MARV coinfection of ERBs modulates MARV shedding dynamics and infectiousness. Although the bats in both experimental cohorts were inoculated with MARV six days following SOSV or KASV, the outcome of the two coinfection experiments were different. Compared to their monoinfected counterparts, SOSV+MARV-coinfected bats exhibited significantly shorter durations of MARV oral shedding and significantly decreased anti-MARV IgG responses, while KASV+MARV-coinfected bats exhibited MARV viremias and oral shedding loads of significantly increased magnitude and duration, as well as significantly increased anti-MARV IgG responses. The distinct evolutionary histories of the coinfecting viruses, SOSV (paramyxovirus that is likely maintained in nature in an ERB-to-ERB transmission cycle) and KASV (orthonairovirus that circulates in nature between ERBs and *O. R. faini* ticks), likely influenced the bats’ immune response to MARV, leading to the observed inhibition of MARV by SOSV pre-infection and the potentiation of MARV shedding by KASV pre-infection.

Individual heterogeneities in pathogen transmission help shape infectious disease dynamics in host populations and can result from variability in host-host interactions and/or host-pathogen interactions [45]. Previous studies across a range of infectious agent-host systems have demonstrated that a minority of infectious hosts are responsible for the majority of infectious agent shedding [46–49] and transmission events [50,51]. Relative to the infected host population, superspreaders are individuals that have more opportunities to infect other hosts, typically through higher contact rates, while supershedders are individuals with higher intensities of infection (i.e., increased magnitude and/or duration of pathogen shedding) [44]. Bat contact rates were not measured in this study; however, daily sampling throughout the MARV infectious period permitted us to determine the effect of MARV coinfection on MARV cumulative shedding, a measure of infectiousness [44], and identify supershedders. Although SOSV+MARV coinfection did not significantly affect overall MARV infectiousness, KASV-MARV-coinfected bats shed 1.2 log_10_TCID_50_eq ml^-1^ more MARV throughout the study than MARV-monoinfected bats. Four of the KASV+MARV-coinfected bats (16.7% of the population) were classified as MARV supershedders and were collectively responsible for 72.5% of the KASV-MARV experimental cohort’s MARV shedding. We speculate that the increased infectiousness of KASV+MARV-coinfected ERBs increases bat-to-bat virus transmission rates and contributes to the explosive nature (versus a lower-level of temporally-sustained MARV transmission in juvenile ERBs) of the biannual spikes of active MARV infection observed in 6-month-old juvenile ERBs at Python Cave and Kitaka Mine, Uganda [27]. Though cumulative shedding is a proxy for infectiousness [44], experimental bat-to-bat transmission studies are ultimately needed to determine the extent to which SOSV+MARV and KASV+MARV coinfection impacts MARV bat-to-bat transmission rates (i.e., Are the number of transmission events from a KASV+MARV-coinfected bat to naïve contact bats greater than the number of transmission events from a MARV-monoinfected bat to MARV-naïve contact bats?) and onward infectiousness (i.e., Do MARV-naïve contact bats housed with KASV+MARV coinfected bats shed more MARV than MARV-naïve contact bats housed with MARV-monoinfected bats?) [46].

We found that SOSV+MARV-coinfected bats had lower anti-MARV IgG responses compared to MARV-monoinfected bats, while KASV+MARV-coinfected bats had higher anti-MARV IgG responses compared to their MARV-monoinfected counterparts. As observed in a previous cohort of captive-bred ERBs prime-challenged with MARV [52], we expect that primary KASV-MARV-coinfection would confer long-term protective immunity against MARV reinfection. Conversely, considering that 2 SOSV-MARV-coinfected ERBs failed to seroconvert to MARV and others had relatively low anti-MARV IgG responses, we speculate that at least some SOSV-MARV-coinfected ERBs might remain susceptible to MARV reinfection as adults. This may partially explain the consistent 2.9% year-round prevalence of active MARV infection observed in adult ERBs despite the explosive biannual spikes in the prevalence of active MARV infection in 6-month-old juveniles [27]. Interestingly, another MARV prime-challenge study utilizing MARV seronegative, wild-caught South African ERBs (rather than captive-bred ERBs) found that primary MARV infection did not generate sterilizing immunity in all bats [53]. Given that multiple paramyxoviruses have been shown to concurrently circulate in this population of South African ERBs [21], it’s possible that the MARV prime-challenged ERBs that were susceptible to MARV reinfection were coinfected with a paramyxovirus during their primary MARV infection.

Approximately half of the known MARV spillover events have been linked to human encroachment on ERB-inhabited subterranean environments (e.g., miners and cave tourists) and likely arose from indirect contact with MARV-contaminated bodily fluids [26,37,38,54–59], such as guano. Though the primary MARV cases for the remaining MARV outbreaks did not report entering subterranean environments or having contact with visibly sick non-human primates prior to becoming ill [59], other plausible bat-to-human MARV transmission mechanisms that may explain how they were infected include indirect contact with MARV-contaminated fruit spats and test-bitten fruit [59,60], as well as direct contact with MARV-contaminated ERB bushmeat [61–63]. Increased MARV infectiousness, as was observed in the KASV+MARV-coinfected supershedders, as well as the KASV+MARV-coinfected bats in general, would likely increase MARV spillover potential by increasing the virus’ chance of overcoming human immunological barriers to infection (i.e., higher viral exposure doses) and increasing the number of spillover opportunities (i.e., prolonged duration of viral shedding and prolonged survival of the virus in the environment due to higher viral deposition loads) [7,60]. The substantial effect that pathogen supershedders can have on zoonotic spillover risk was highlighted in a previous ecological modeling study [64] that predicted environmental contamination by *Escherichia coli* O157-supershedding cattle increases the risk of human infection by 100─1000-fold [44].

While our data suggest that KASV+MARV coinfection of ERBs can impact bat-to-bat transmission dynamics and spillover potential, collection of additional ecological and experimental data (e.g., mass of guano produced/day/bat, MARV decay rate in feces and on fruit, etc.) are needed to parametrize dose-response models [64] of MARV shedding from ERBs and investigate the effect that environmental contamination by MARV supershedder ERBs has on the relative risk of MARV spillover. Additionally, fine-scale longitudinal data on MARV, SOSV, and KASV infection in ERBs, as well as age-stratified ERB contact rates, are needed to parameterize epidemiological models of MARV transmission to explore how coinfection shapes temporal spillover risk. Further experimental studies are also needed to assess the impact that inoculation order (i.e., MARV inoculation followed by SOSV or KASV inoculation, and simultaneous SOSV+MARV and KASV+MARV inoculation), inoculation timing (i.e., varying time intervals between inoculation), inoculation with SOSV+KASV+MARV, and inoculation with microorganisms across different taxonomic ranks that ERBs host (e.g., bacteria [65], nematodes [66], and protozoa [67], etc.) has on MARV infectiousness. Lastly, it is important to emphasize that wild ERBs are likely subject to multiple ecological stressors besides coinfection such as stress [68–71], pregnancy, mating, temperature fluctuations, and predation that may further modulate MARV shedding in SOSV+MARV- and KASV+MARV-coinfected ERBs.

Collectively, our results demonstrate that, depending on the specific coinfecting virus, MARV coinfection of ERBs differentially modulate MARV infectiousness and anti-MARV IgG responses and implicate coinfection as playing a critical role in bat-to-bat MARV transmission dynamics and spillover potential.

## Materials and methods

### Ethics statement

A total of 52 captive-bred juvenile ERBs (5─7 months of age) from the ERB breeding colony were used in this study [28]. All animal procedures were approved by the Institutional Animal Care and Use Committee (protocols SCHBATC3203-A1 and SCHBATC3313-A2) at the US Centers for Disease Control and Prevention (CDC), an Association for Assessment and Accreditation of Laboratory Animal Care accredited research facility. As infectious MARV (Tier 1 Select Agent) was used in this study, all procedures conducted with infectious virus or virus-infected animals were performed at the CDC under biosafety level 4 (BSL-4) containment in compliance with Select Agent Regulations [72].

### ERB husbandry and monitoring

ERBs were acclimated to the BSL-4 environment for at least 5 days prior to study initiation. The bats were housed according to group in NHP-sized (or larger) cages maintained within bio-flow isolators with HEPA-filtered inlet and exhaust air supplies (Duo-Flow Mobile Units, Lab Products Inc., Seaford, DE) in a climate-controlled animal area with a 12 h day/12 h night cycle. Bats were provided with their body mass daily in fresh fruit sprinkled with a fruit bat-optimized protein supplement and received water *ad libitum*. Animal care staff and/or study investigators monitored the bats daily for clinical signs of disease.

### Experimental design

The 52 ERBs were assigned to one of two experimental cohorts: 1) SOSV-MARV (n=26 bats; work performed in October and November 2021) or 2) KASV-MARV (n=26 bats; work performed in February and March 2023). Bats in the SOSV-MARV experimental cohort were divided into 3 groups: 1) NEG CO (1 male and 1 female), 2) SOSV+MARV (7 males and 5 females), and 3) MARV only (7 males and 5 females). Bats in the KASV-MARV experimental cohort were divided into 3 groups: 1) NEG CO (1 male and 1 female), 2) KASV+MARV (7 males and 5 females), and 3) MARV only (7 males and 5 females).

At -6 days post MARV inoculation, NEG CO bats and SOSV+MARV bats in the SOSV-MARV experimental cohort were subcutaneously inoculated with 0.25 mL of sterile media or 4.0 log_10_TCID_50_ of recombinant SOSV (RecSosuga-wt isolate, ERB spleen+1 passage, Vero E6+2 passages; mycoplasma-free, GenBank Accession Number PP750364), respectively. Six days later at 0 days post MARV infection, the NEG CO bats were subcutaneously inoculated 0.25 mL of sterile media, while the SOSV+MARV bats and MARV only bats were subcutaneously inoculated with 4.0 log_10_TCID_50_ of MARV (Uganda 371Bat2007 isolate; Vero E6+2 passages; mycoplasma-free; GenBank Accession Number: FJ750958), respectively. Likewise, at -6 days post MARV infection, NEG CO bats and KASV+MARV bats in the KASV-MARV experimental cohort were intradermally inoculated with 0.10 mL of sterile media or 4.0 log_10_TCID_50_ of KASV (UGA-Tick-20170128 isolate; Vero E6+2 passages; mycoplasma-free; sequence identical to GenBank Accession Numbers MT309090, MT309094, and MT309097 [Vero E6+1]), respectively. Six days later at 0 days post MARV infection, the NEG CO bats were subcutaneously inoculated with 0.25 mL of sterile media, while the KASV+MARV bats and MARV only bats were subcutaneously inoculated with 4.0 log_10_TCID_50_ of MARV. All inoculations occurred while the bats were under vaporized isoflurane anesthesia and were administered in the subcaudal abdominal region. Subcutaneous inoculation of bats with SOSV [31] and MARV [28,41] were performed to mimic virus transmission by the bite of an infectious ERB, while intradermal inoculation of bats with KASV was performed to mimic virus transmission by the bite of an infectious *O. (R.) faini* tick [41].

Bats were manually restrained during sample collection by a “holder” donning bite-resistant gloves. Whole blood was collected from bats in both experimental cohorts prior to the initiation of the study. For bats in the SOSV-MARV experimental cohort, whole blood was collected at -5, -3, -1-, 3-, 5-, and 7-days post MARV inoculation (not a primary shedding route for SOSV or MARV and therefore collected blood samples on an intermittent basis only), oral swabs (primary shedding route for MARV), temperatures, and rectal swabs (primary shedding route for SOSV) were collected daily through the end of the study (i.e., 18 days post MARV inoculation; 24 days post SOSV inoculation; real-time monitoring of viral shedding allowed us to end the study once viral shedding from all bats had ceased), and weights were taken weekly. For bats in the KASV-MARV experimental cohort, blood (primary shedding route for KASV), oral swabs (primary shedding route for MARV), temperatures, and rectal swabs were collected daily through the end of the study (i.e., 21 days post MARV inoculation; 27 days post KASV inoculation; real-time monitoring of viral shedding allowed us to end the study once viral shedding from all bats had ceased); and weights were taken weekly. At the end of the study, the bats were either euthanized by an overdose of isoflurane followed by cardiac exsanguination or transferred to another study.

Whole blood (10 µL placed in 500 µL MagMax lysis buffer for RNA extraction; 21 µL placed in 500 µL masterplate diluent for serology and then frozen) was collected from the cephalic vein using a sterile lancet (C&A Scientific, Manassas, VA); polyester-tipped applicators (Fisher Scientific, Grand Island, NY) were used to swab the oral mucosa (placed in 500 µL MagMax lysis buffer for immediate RNA extraction); rectal swabs were obtained opportunistically at the time rectal temperatures were taken by repurposing the plastic thermometer probe cover (MABIS Healthcare, Waukegan, IL; placed in 500 µL MagMax lysis buffer for immediate RNA extraction); weights were taken using a small cotton weigh bag and digital scale.

Blood was used to quantitate viremia (virus-specific RNA by reverse transcriptase-quantitative polymerase chain reaction [RT-qPCR]) and antibody responses (anti-virus-specific IgG by indirect enzyme-linked immunosorbent assay [ELISA]); oral and rectal swabs were used to quantitate virus shedding (virus-specific RNA by RT-qPCR).

### RNA extraction and RT-qPCR

RNA was extracted from whole blood, oral swabs, and rectal swabs using the MagMAX Pathogen RNA/DNA Kit (Thermo Fisher Scientific, Waltham, MA) on the KingFisher Apex Dx (Thermo Fisher Scientific). Reverse-transcribed MARV, SOSV, KASV and ERB beta-2-microglobulin (B2M; housekeeping gene) RNA was detected on the CFX Opus Real-Time PCR System (Bio-Rad, Hercules, CA) using the Luna Probe One-Step RT-qPCR 4X Mix with UDG (New England BioLabs; S1 Table) and either the M1-S1-B1 (samples collected from bats in the SOSV-MARV experimental cohort) or M1-K2-B1 (samples collected from bats in the KASV-MARV experimental cohort) multiplex RT-qPCR assay (S2 Table). It is important to note that the multiplex RT-qPCR assays were designed to detect RNA from ERBs experimentally infected with a single isolate of SOSV (GenBank Accession No.: PP750364), KASV (GenBank Accession No.:MT309090), and MARV (GenBank Accession No.: FJ750958) and should not be used to detect other virus sequences. Relative SOSV, KASV, and MARV TCID_50_eq ml^-1^ (blood, oral, and rectal specimens) were interpolated from standard curves generated from serial dilutions of the titrated virus isolates spiked into appropriate biological specimens.

### Serology

Whole blood collected from bats prior to and at the end of the study was tested for anti-MARV nucleoprotein (NP) IgG (SOSV-MARV and KASV-MARV experimental cohorts), anti-SOSV IgG (SOSV-MARV experimental cohort), and anti-KASV IgG (KASV-MARV experimental cohort).

Wells of 96-well ELISA plates were coated (100 µL) with MARV NP (3 ng/well), KASV infectious-based lysate (1:2000), or SOSV infectious based lysate (1:500) and corresponding wells were coated with an equivalent concentration/dilution (diluent: PBS containing 1% thimerosal) of Reston virus (RESTV) NP, uninfected Vero E6 lysate, and uninfected Vero E6 lysate, respectively. After incubation overnight at 4 °C, the plates were washed with PBS containing 0.1% Tween-20 (PBS-T) and 100 µL of serum diluent (PBS containing 5% skim milk and 0.1% tween-20) was added to each well of the plate. After 10 min, 33 µL of a 21:521 dilution of gamma-irradiated whole blood pre-diluted in masterplate diluent (PBS containing 5% skim milk powder, 0.5% tween-20 and 1% thimerosal) was added to the first well of the plate and 4-fold serial dilutions, ranging from 1:100─1:6,400, were performed. Following a 1 hr incubation at 37 °C, the plates were washed with PBS-T and 100 µL of a 1:11,000 (anti-MARV NP IgG and anti-SOSV IgG assays or 1:20,000 (anti-KASV IgG assay) dilution of goat anti-bat IgG conjugated to horseradish peroxidase (Bethyl Laboratories, Montgomery, TX; Cat#: A140-118P, Lot#: A140-118P-17) in serum diluent was added to the plates. After incubation for 1 hr at 37 °C, the plates were washed with PBS-T, 100 µL of the Two-Component ABTS Peroxidase System (KPL, Gaithersburg, MD) was added, and the plates were allowed to incubate for 30 min at 37 °C. The plates were then read on a microplate spectrophotometer set at 410 nm.

To reduce non-specific background reactivity, adjusted optical density (OD) values were calculated by subtracting the ODs at each 4-fold dilution of wells coated with RESTV NP or uninfected control antigen lysate from ODs at corresponding wells coated with MARV NP, SOSV lysate, or KASV lysate, respectively. The adjusted sum OD value was determined by summing the adjusted OD values at each four-fold serial dilution and then linearly transforming the values using the min-max normalization method. The cut-off values for assay seropositivity were determined by calculating the mean adjusted sum OD value plus 3─5 standard deviations (SDs) of MARV, SOSV, and KASV naïve ERBs (38─76 specimens depending on assay).

### Data and statistical analyses

Each comparator group was comprised of 12 juvenile bats. The number of bats per group was based on the reproductive capacity of the ERB breeding colony, the number of bats a team could safely handle daily, the available animal space in the BSL-4 lab, and a prior experimental study showing that the mean cumulative quantity of virus shed from the oral mucosa of 12 MARV-infected ERBs was 4.7 log_10_TCID_50_ ml^-1^ (+/- 5.1 SD) [41]. Power calculations (power=0.8, alpha=0.05) based on this prior MARV cumulative oral shedding data revealed that we would need a minimum of 12 bats per group to demonstrate a statistically significant difference in cumulative oral shedding. Bat groups were sex-matched as described above, investigators were not blinded during the study, and no bats or individual data points were excluded from the analyses.

Excel (Microsoft 365, Redmond, WA) was used to manage data and GraphPad Prism 10 (GraphPad, La Jolla, CA) was used to perform statistical analyses and generate figures. MARV peak loads and the duration of viral shedding was determined for each bat according to experimental cohort (SOSV-MARV and KASV-MARV), bat group (SOSV+MARV and MARV only; KASV-MARV and MARV only), and sample type (blood [KASV-MARV experimental cohort only], oral swab, and rectal swab). To assess MARV infectiousness, cumulative viral shedding loads were calculated for each bat according to experimental cohort and bat group by summing viral loads detected in blood (KASV-MARV experimental cohort only; blood was collected from bats in the SOSV-MARV experimental cohort to assess MARV viremia at 4 time points only), oral swabs, and rectal swabs through the duration of the study (1─18 DPI for the SOSV-MARV experimental cohort; 1─21 DPI for the KASV-MARV experimental cohort). Using the approach of Jankowski et al. [47], bats were classified as supershedders if they shed MARV at loads ≥ the 80^th^ percentile.

The Shapiro-Wilks test was used to determine if peak viral load, duration of viral shedding, and cumulative viral shedding load datasets followed a normal or lognormal distribution. If datasets were normally-distributed, then unpaired t-tests were used to determine if parameter means differed significantly between virus coinfected and monoinfected bat groups. If datasets were log-normally-distributed, they were log-transformed before using unpaired t-tests to determine if parameter geometric means differed significantly between virus coinfected and monoinfected bat groups. If datasets did not follow a normal or lognormal distribution, then non-parametric Mann-Whitney U tests were used to determine if parameter mean ranks differed significantly between virus coinfected and monoinfected bat groups. All P values were two-tailed and P< 0.05 was considered statistically significant. Textual data are presented as mean/geometric mean ± SD/geometric SD (GSD), and graphical data are presented as mean/geometric mean with all individual points to show the data distribution and range. Each bat represents an individual biological replicate.

## Supporting information

S1 Table Thermocycling conditions for the multiplex reverse transcription quantitative real-time polymerase chain reaction assays. (DOCX)

S2 Table Multiplex reverse transcription real-time quantitative polymerase chain reaction assays. (DOCX)

S1 Fig Weights and body temperatures of bats assigned to the SOSV-MARV and KASV-MARV experimental cohorts. (A) Percent weight change from baseline for bats in the SOSV-MARV experimental cohort, (B) percent weight change from baseline for bats in the KASV-MARV experimental cohort, (C) body temperatures for bats in the SOSV-MARV experimental cohort, and (D) body temperatures for bats in the KASV-MARV experimental cohort. Symbols in a-d represent individual bats and solid lines represent arithmetic means.
(TIF)

S1 Data Data used to generate Figs 2–7 and S1 Fig. (XLSX)
