# Supplementary material for: Modeling natural coinfection in a bat reservoir shows modulation of Marburg virus shedding and spillover potential
Source: PLoS Pathog. 2025 Mar 17;21(3):e1012901. doi: 10.1371/journal.ppat.1012901 (PMC11978059; doi:10.1371/journal.ppat.1012901)
Supplement: S1 Table — (DOCX) [file ppat.1012901.s001.docx]

| **Step** | **Temperature** | **Time** | **Cycles** |
| --- | --- | --- | --- |
| Reverse Transcription | 55°C | 10 min | 1 |
| Initial Denaturation | 95°C | 1 min | 1 |
| Denaturation | 95°C | 10 sec | 40 |
| Extension | 60°C | 30 sec (+plate read) |  |
