# Supplementary material for: Modeling natural coinfection in a bat reservoir shows modulation of Marburg virus shedding and spillover potential
Source: PLoS Pathog. 2025 Mar 17;21(3):e1012901. doi: 10.1371/journal.ppat.1012901 (PMC11978059; doi:10.1371/journal.ppat.1012901)
Supplement: S2 Table — (DOCX) [file ppat.1012901.s002.docx]

| Assay | Gene target | Name | Sequence (5’ to 3’) | Final concentration |
| --- | --- | --- | --- | --- |
| M1-S1-B1 | MARV NP | MF1 | AAGTTGCTAGTTTCAAGCAGGCGTTGA | 0.4 µM |
|  |  | MR1 | CCATGCTCGAGGTTGTTAATCCCTGATAAA | 0.4 µM |
|  |  | MP1 | 56-FAM-AACCCGTGC-ZEN-AAATGGTGCGTATTCTCCATG-3IABkFQ |  |
|  | SOSV NP | SF1 | TTATGCAATGGGAATCGGAAGTGTCCAAGA | 0.4 µM |
|  |  | SR1 | CCCCAATTGGTAAAATGCAGCATTCAAGTA | 0.4 µM |
|  |  | SP1 | 5HEX-CTGCCAAAT-ZEN-GCATAGCCTCGCATTTGAGGA-3IABkFQ | 0.2 µM |
|  | ERB B2M | BF1 | CCTGGACGGCGAGCCACGTACTCCAAA | 0.2 µM |
|  |  | BR1 | TGGGTGGAATCCATACACATAGCAGTTTA | 0.2 µM |
|  |  | BP1 | 5Cy5-CTCTGCTGG-TAO-ATGCCGTGAATAAACCTGAAC-3IAbRQSp | 0.2 µM |
| M1-K2-B1 | MARV NP | MF1 | AAGTTGCTAGTTTCAAGCAGGCGTTGA | 0.4 µM |
|  |  | MR1 | CCATGCTCGAGGTTGTTAATCCCTGATAAA | 0.4 µM |
|  |  | MP1 | AACCCGTGCAAATGGTGCGTATTCTCCATG |  |
|  | KASV NP | KF2 | CCTGAGTTCAAGGTCTGGCACGACAAGTA | 0.4 µM |
|  |  | KR2 | CCCAGAGCAAGTCTGGTAAGCCAGAA | 0.4 µM |
|  |  | KP2 | 5HEX-CAGCCTCAG-ZEN-GCAGGTTGGACTTCAAGTCAT-3IABkFQ | 0.2 µM |
|  | ERB B2M | BF1 | CCTGGACGGCGAGCCACGTACTCCAAA | 0.2 µM |
|  |  | BR1 | TGGGTGGAATCCATACACATAGCAGTTTA | 0.2 µM |
|  |  | BP1 | 5Cy5-CTCTGCTGG-TAO-ATGCCGTGAATAAACCTGAAC-3IAbRQSp | 0.2 µM |

MARV: Marburg virus, NP: Nucleoprotein, SOSV: Sosuga virus, ERB: Egyptian rousette bat, B2M: Beta-2-microglobulin, KASV: Kasokero virus; 56-FAM: 5' 6-fluorescein modification, ZEN: proprietary internal quencher, 3IABkFQ: 3’ Iowa Black fluorescent quencher, 5 HEX: 5’ hexachlorofluorescein, 5Cy5: 5’ Cyanine 5, TAO: proprietary internal quencher; 3IAbRQSp: 3’ Iowa Black red quencher.
