# Supplementary figures and images for: Modeling natural coinfection in a bat reservoir shows modulation of Marburg virus shedding and spillover potential
Source: PLoS Pathog. 2025 Mar 17;21(3):e1012901. doi: 10.1371/journal.ppat.1012901 (PMC11978059; doi:10.1371/journal.ppat.1012901)

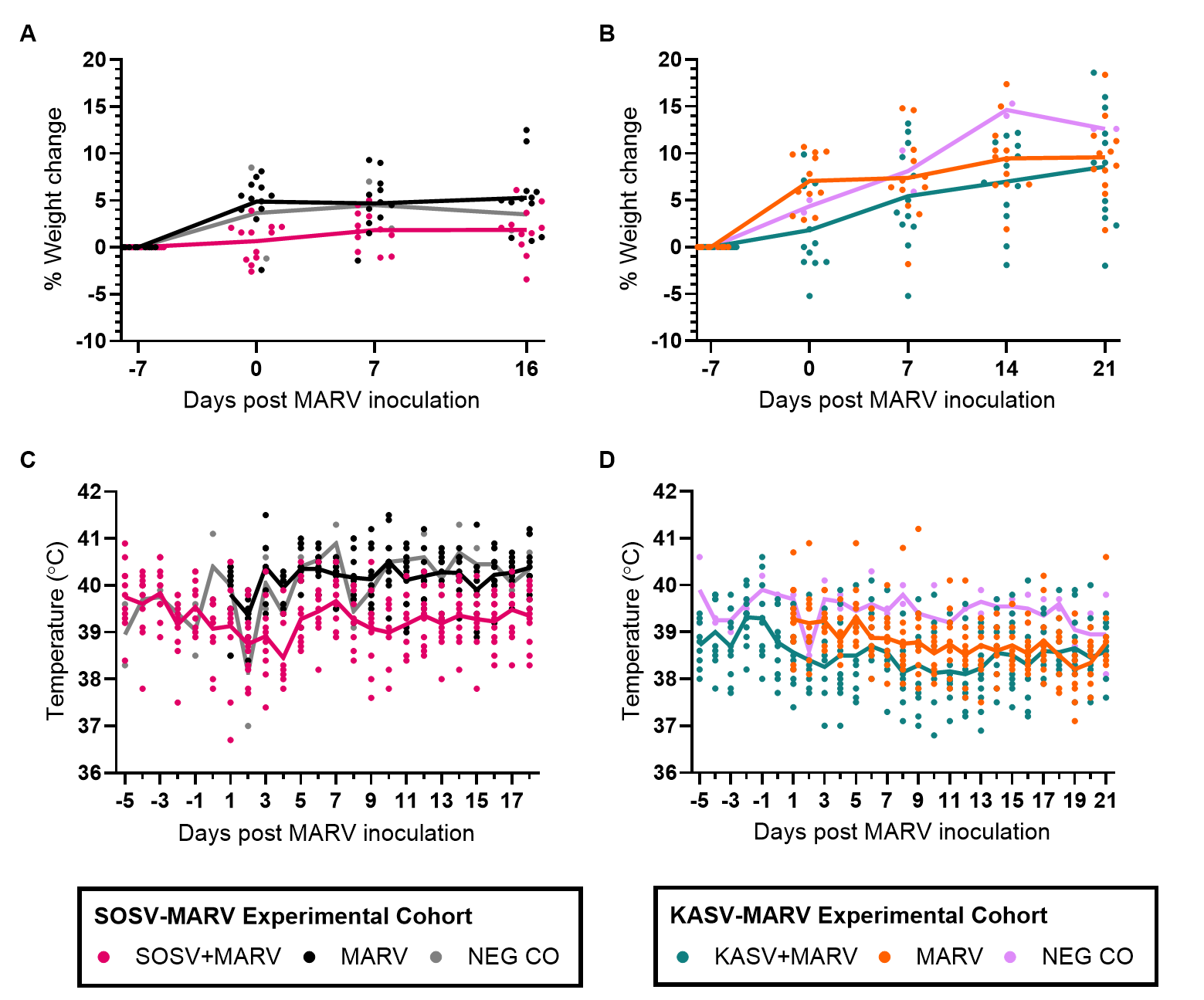

Supplement: S1 Fig — (A) Percent weight change from baseline for bats in the SOSV-MARV experimental cohort, (B) percent weight change from baseline for bats in the KASV-MARV experimental cohort, (C) body temperatures for bats in the SOSV-MARV experimental cohort, and (D) body temperatures for bats in the KASV-MARV experimental cohort. Symbols in a-d represent individual bats and solid lines represent arithmetic means. (TIF) [file ppat.1012901.s003.tif]
